# Diagnostic Reasoning across the Medical Education Continuum

**DOI:** 10.3390/healthcare2030253

**Published:** 2014-07-15

**Authors:** C. Scott Smith, William Hill, Chris Francovich, Magdalena Morris, Bruce Robbins, Lynne Robins, Andrew Turner

**Affiliations:** 1 Department of Medicine, VA Medical Center, Boise, ID 83702, USA; E-Mails: tatlnd@aol.com (W.H.); francovich@gonzaga.edu (C.F.); magnan@spro.net (M.M.); 2 Behavioral Informatics and Medical Education, University of Washington, Seattle, WA 98195, USA; E-Mail: lynner@u.washington.edu; 3 Leadership Studies, Gonzaga University, Spokane, WA 99258, USA; 4 Department of Nursing, Eagle Gate College, Murray, UT 84123, USA; 5 English Department, Boise State University, Boise, ID 83725, USA; E-Mail: brobbins@boisestate.edu; 6 Santa Rosa Community-based Outreach Clinic, VA Medical Center, San Francisco, CA 95403, USA; E-Mail: Andrew.Turner2@va.gov

**Keywords:** undergraduate medical education, graduate medical education, emotional stress, narrative medicine, diagnostic reasoning, professional competence, curriculum, personal narratives

## Abstract

We aimed to study linguistic and non-linguistic elements of diagnostic reasoning across the continuum of medical education. We performed semi-structured interviews of premedical students, first year medical students, third year medical students, second year internal medicine residents, and experienced faculty (ten each) as they diagnosed three common causes of dyspnea. A second observer recorded emotional tone. All interviews were digitally recorded and blinded transcripts were created. Propositional analysis and concept mapping were performed. Grounded theory was used to identify salient categories and transcripts were scored with these categories. Transcripts were then unblinded. Systematic differences in propositional structure, number of concept connections, distribution of grounded theory categories, episodic and semantic memories, and emotional tone were identified. Summary concept maps were created and grounded theory concepts were explored for each learning level. We identified three major findings: (1) The “apprentice effect” in novices (high stress and low narrative competence); (2) logistic concept growth in intermediates; and (3) a cognitive state transition (between analytical and intuitive approaches) in experts. These findings warrant further study and comparison.

## 1. Introduction

Our study aimed to explore differences in diagnostic reasoning over the continuum from premedical students to experienced faculty. The role of narrative memory structures and “promising-primed” ecological decision making (described below) were expected to be critical for expert diagnostic reasoning. Our interest began with several puzzling phenomena that have been identified in studies of clinical reasoning. Researchers have discovered that residents (intermediate level learners) generate more facts and rules than students or faculty when explaining their approach to clinical problems, despite the fact that faculty have more experience [1,2]. Successful experts do not generate hypotheses faster or in greater number, they simply generate better initial hypotheses [3,4,5]. However, experts revert to slower analytical approaches outside of their field of expertise [4]. Clinical reasoning has also been shown to be highly context dependent; that is, diagnostic decision making is better when the context resembles that in which the knowledge will be used [3,6].

One prominent model of clinical reasoning, the encapsulation theory of Bordage and Schmidt [7,8,9], suggests that practitioners transition from reduced knowledge, to causal networks, to abridged networks, and then to highly efficient compiled structures such as production rules [4], semantic networks [10], and prototypes [11]. With experience, memory is organized into relational groups or “chunks” that facilitate retrieval [12,13]. Search strategies within these groups, encoded as schemas [14] or scripts [15], are generally subconscious and automatic.

Under this theory novices exhibit simple networks of cause-effect relationships and concrete concepts. For intermediates, clinical experience leads to coherent, abridged networks based on abstract clinical concepts such as “sepsis”. This elaborated knowledge has rich propositional structures that are highly interconnected by semantic associations and learners at this level use more abstract terms. With significantly more clinical experience, experts with compiled knowledge have organized formal and clinical knowledge into illness scripts (prototypes) or instance scripts (explicit memories of specific patients) for highly efficient storage and retrieval. These memory structures are generally subconscious and may be hard for experienced clinicians to articulate. This model of diagnostic reasoning in experts assumes that clinical decision making is solely a process “in-the-head” acting on external stimulus information.

However, the organism/environment relationship may not be as crisply delineated as the standard clinical reasoning model posits. Some neuroscientists have entertained the possibility that cognition is extended into the world [16]. In fact, the field of ecological psychology assumes this dynamic, co-determined organism/environment relationship [17,18]. From this perspective, the environment itself (including the patient) contains critical information. We have evolved preconscious information-seeking behaviors that access and utilize these information resources [17,18]. As an example, subconscious saccadic eye movements may be drawn to cyanotic lips and wrinkles in a patient complaining of shortness of breath, tacitly searching for a significant smoking history. This ecological perspective is becoming more important in medical education research [19,20].

Another hint that something more complex may be going on is Bereiter and Scardemalia’s concept of “promisingness” [21]. They found that experts automatically recognize more promising paths by simultaneously attending to goals, their own capabilities and feelings, and complex informational connections. This skill depends on the integration of prior knowledge with experience. Klein has explored this phenomenon by studying decision making in real world contexts and found that experts use “recognition-primed” decision making [22]. In complex situations, tacit contextual information may trigger reflexive action in the expert that is difficult for them to describe or even understand. For instance, a fire chief leads his team into a kitchen fire in a single-story house. They spray the fire with water but it unexpectedly flares back up. He has a “sixth sense” and orders his men out just before the floor collapses. After extensive debriefing he realized that the fire was too quiet, suggesting to him that there was noise insulation between himself and the source (an unexpected basement), which then makes sense of the lack of efficacy when water was sprayed into the kitchen [22]. Both promisingness and recognition-primed decision making suggest that experts use rapid processes of matching tacit information in the environment to the most coherent experience-based memory structure. These “promising-primed” explanations, such as pneumonia in the smoker with shortness of breath, generate familiar physical, cognitive, and emotional reactions in us because they coherently explain the data and just “feel right”. Combined with information-seeking behaviors, this promising-primed ecological decision making may be an important feature of expertise.

Recent dual process models of decision making such as prospect theory [23] or fuzzy trace theory [24] suggest this is true. Much of human categorization and decision making begins with preconscious categories (intuition or “gist”). These categories are based in an emotional tone and represented as vague, qualitative rules such as “chemotherapy is poison” [24]. These preconscious categories then may be endorsed, adjusted (often imperfectly) or replaced by slow, rule-based, linear categorization schemes (rule-based processing). Irrelevant but highly accessible features may lead to heuristic errors [23]. The dopaminergic system focuses attention on discrepancies between expectations and actual experience. Over time, this “dopaminergic tuning” leads to richer, more accurate cognitive models [25,26]. Experienced decision makers rely increasingly on preconscious categorization, using fewer dimensions of information and processing it more crudely (all/none). This paradoxically leads to better discrimination [24].

One particularly robust human memory structure is narrative. Story structure has deep roots in primate and human evolution, producing selective advantage by focusing attention, fostering group collaboration, exploring intentions and motives for action, moral instruction, and predicting causation [27]. Stories provide for the human equivalent of “massive parallel processing”, whereby we may act not just from our own experience but from that of the collective. Narrative is known to play a crucial role in the organization of preconscious gestalts and rule-based, linear processing [28]. Stories identify agents with their character traits; relay their goals, intentions and beliefs; and seek the coherence that allows causal reasoning. Psychological studies of categorization in complex, ill-structured domains such as juror decision making confirm that narrative structure is imposed on ill-structured data as it is collected, and that formal category assignment is made by the “best story match” [29]. These stories may be organized in family resemblance groupings to facilitate retrieval (like “small worlds” [12] or “logical competitor sets” [13]), and focus on critical cues that quickly identify the best story (like crucial tests in schemes [14] or scripts [15]).

## 2. Experimental Section

This cohort study was approved by the institutional review boards (human subjects) at all participating sites. Because of the spectrum of development we expected, our methods reflect two broad considerations. First, we needed to identify a data collection system capable of elucidating both formal (didactic) and experience-based (clinical) learning. Second, we needed to choose analytical methods capable of identifying both rational (conscious, logical, algorithmic) and intuitive (preconscious, emotion-based, pattern recognition) approaches to diagnosis. For data collection, we chose semi-structured interviews with trigger questions that would generate experiences, knowledge, reflection, and memories. We chose propositional analysis and concept mapping to explore rational diagnosis and grounded theory and a measure of emotional tone to explore aspects of intuitive diagnosis.

The interviews were performed by an experienced qualitative researcher on ten each of premedical students (PM), first year medical students (MS1), third year medical students (MS3), second year internal medicine residents (R2), and experienced internal medicine faculty (F). For each of three diseases (congestive heart failure, emphysema, and pneumonia) the following four trigger questions were asked and the answers were audio taped:

- If someone complained of shortness of breath, how would you figure out that the cause was [*insert disease*]?
- What do you believe are the critical elements for making that diagnosis?
- What helped or hindered you in learning this?
- Can you remember anyone (an acquaintance, family member, or patient) with this disease?

Interviews took approximately 30–45 min each.

Transcripts were blinded to learner level and were analyzed by two teams of researchers. One seasoned clinician performed propositional analyses and used these to create concept maps. Any “IF➔THEN” assertion such as “congestive heart failure causes lower extremity edema” was identified as a single proposition. These were identified and counted. Then, propositions were arranged into concept maps, which detailed the branching hierarchical structure where the logical relationship between concepts could be articulated in linking phrases such as “gives rise to”, “results in”, “is required by”, or “contributes to” [30]. The number of connections between concepts in each concept maps was used as a measure of conceptual robustness. Propositions and concept maps were validated by the data collection team for accuracy.

Another team used grounded theory to identify and negotiate salient categories in the data and code passages in the transcripts with these categories. Each individual’s response to each question was a unit of analysis. These researchers independently read and reread a subset of 5% of the transcripts as a training set, identifying categories that explained the subject’s diagnostic approach to the disease. Each category was defined with necessary and sufficient conditions and exemplified with text examples. This team met several times to negotiate categories and definitions until agreement was reached for the training set. Discrepancies were adjudicated by the principal investigator. The final categories were further validated by member checking for accuracy and completeness with all groups. All transcripts were then coded with the consensual set of categories.

Additionally, another intent was to separate “instance scripts” (explicit patient care memories) from “illness scripts” (prototypes) [11] to explore memory structures across the continuum of expertise. We grouped responses to the question “can you remember anyone with this disease” according to whether they were episodic (personal perspective, instance script) or semantic (factual, illness script) memories. Semantic memories were further divided into explanations, predictions, prototypes, and qualifiers because of important differences observed in the coding (for instance, whether factual memory represented a rule, a common presentation, or a cusp case).

During the interviews, a process observer recorded emotional tone with a validated tool based on facial expression and body language (FACES) [31]. This instrument has six gradations anchored with descriptors (such as tense, alert, aroused, excited) that range between unpleasant and pleasant, producing a range of scores between −3 and +3.

Once these analyses were carried out, the learning level of each transcript was un-blinded. The summary concept maps were created, and those maps and grounded theory categories were explored for each learning level.

## 3. Results

### 3.1. Novices: The Apprentice Effect

**Figure 1:**
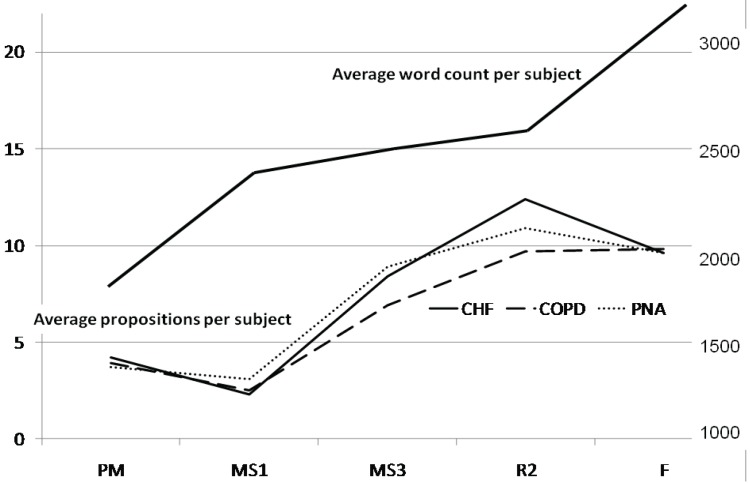
Average word count and propositions per subject.

MS1s talked relatively more and said less than any other group (Table 1 and Figure 1). The average number of propositions and the percentage of words dedicated to propositions for each disease group was the lowest for MS1s and peaked in the R2 year. At the same time, the word count per transcript steadily increased with experience level. In addition, there was evidence that, with more experience, participants moved from pathophysiologic descriptions to higher level abstractions for all diseases. For example, in heart failure “fluid buildup”, seen in PMs, MS1s, and MS3s, became “volume overload” thereafter. “Lung pain” (PMs) and “chest pain” (MS1s) in pneumonia became “pleuritic pain” thereafter. “Improper capillary gas exchange” in emphysema for PMs and MS1s became “hypoxemia” and “hypoventilation” thereafter.

**Table 1:** Average numbe of propositions per disease group and number of concept connections in concept maps by training level.

Training Level	Average # of Propositions Per Disease Group (Standard Deviation)	Percentage of Total Words that Are Propositions	Number of Concept Connections in Summary Concept Map
PM	4.2 (2)	2.7%	30
MS1	2.3 (1.8)	1.4%	25
MS3	8.4 (3.1)	5.5%	34
R2	12.4 (5.4)	7.8%	56
F	9.6 (4.2)	4.3%	35

PM = premedical student; MS1 = first year medical student, MS3 = third year medical student, R2 = second year internal medicine resident, F = experienced medical faculty.

PM—premedical student; MS1—first year medical student; MS3—third year medical student; R2—second year internal medicine resident; F—faculty member.

**Figure A1:**
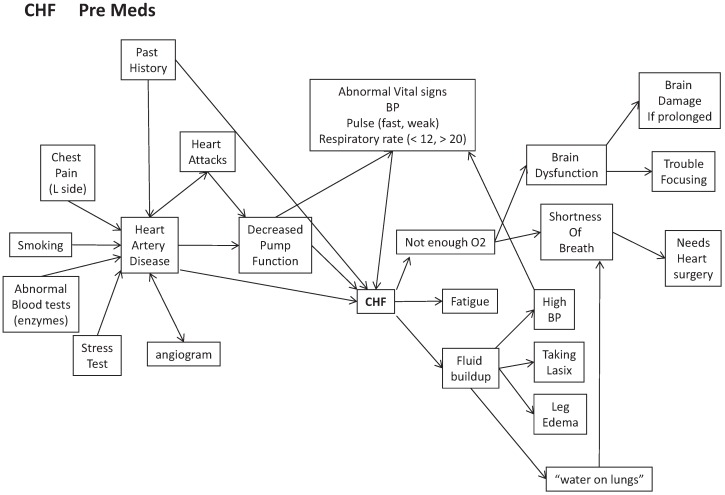
Premedical students.

Concept maps for each training level are shown in the Appendix (Figure A1–Figure A5). These also showed that the number of concept connections was lowest for MS1s and highest for R2s (Table 1). There was a qualitative difference between trainees (at all levels) and experienced faculty. Other than MS1s, trainees showed increasingly complex rule structures for making diagnoses. Faculty, on the other hand made their diagnoses by matching patient presentation to a prototypical story.

MS1s may have learned to distrust personal experience as a guide. For instance, PM’s mentioned non-traditional sources of information such as television, whereas MS1’s did not, mentioning only formal curricular elements (lectures and reading). MS1’s frequently mentioned the belief that some test might make the diagnosis, which might represent a strong desire for concreteness, discomfort with uncertainty, or both. For instance, when asked what helped or hindered in learning a particular pathology:

**Example 1 **(PM) “… on TV if they had specials on Oprah or on any other shows”.
**Example 2 **(PM) “… with congestive heart failure, um… I’d check for weight, check cholesterol levels, and this is mainly from ‘House’ [a TV show]”.
**Example 3 **(MS1) “I feel like labs would help a lot… x-rays or CT scans”.
**Example 4 **(MS1) “There might be some kind of a characteristic signature in the lab values that would lead you to that [diagnosis]”.
**Example 5 **(MS1) “Shortness of breath you can tell upon, just observation of the patient. How to determine that it’s due to congestive heart failure? Um, I don’t know the exact process, or which exams, or which laboratory I would order.”

The grounded theory team identified 17 categories in the transcript data that appeared to be independent elements (Table 2). Inter-rater scoring agreement was 50% at the beginning of the training set and improved to 92% by the end of the training set. Agreement remained 92% across the remainder of the data set.

**Table 2:** Grounded theory categories and definitions.

Category Title	Definition
**A Story**	Where the participant relates personal experience related to a specific other(s). Each specific other is another story.
**Cue Generation**	A list of cues generated to explain or define diagnosis.
**Disconfirming Cue**	The absence of an expected cue or the presence of a cue that negates a diagnosis.
**Experience**	Referral to personal experience by participant as a guide for diagnostic thinking.
**Group Identity**	Where participants refer to or include a “we” or group identifier to their language.
**Logic**	Where some effort is made to formalize an argument for a diagnosis.
**Patient History**	Where the participant explicitly refers to patient history to help them make a diagnosis.
**Physical Exam**	Explicit specific reference to the physical exam as the source of information for the diagnosis.
**Recognition of Case Complexity**	Where there is reference to two or more target diagnoses or other complex factors complicating accurate response to the diagnostic question.
**Rule Based Response**	Participants rely on a general rule or algorithm to respond completely to the question.
**Social Context**	Commentary or conversation around the broader implications of a diagnosis. Intersection of diagnosis and wider social world.
**Struggling with Disease Definition**	Where the participant appears to struggle with the definition or list of disease or diagnosis.
**Symptoms**	Participant refers to the symptoms associated with an ailment.
**Technical Definition**	Using any terminology most lay persons wouldn’t know.
**Tests**	Participant language that suggests or refers to some sort of test to determine diagnosis.
**Treatment**	A referral by the participant to a medical intervention.

**Figure 2:**
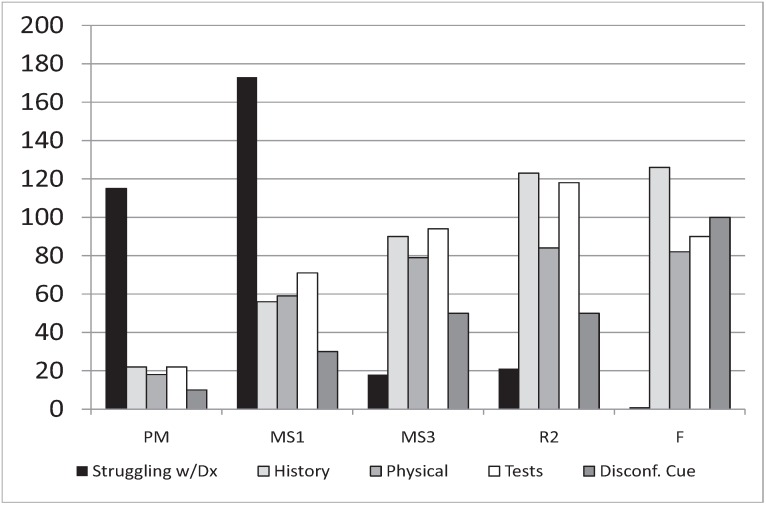
Average number of text units by training level for five key categories: struggling with disease definition, history, physical exam, tests, and disconfirming cue.

One key category identified is *struggling with disease definition*, “where the participant appears to struggle with the definition or list of disease or diagnosis”. This demonstrated a peak in the MS1 year (Figure 2).

PM = premedical student; MS1 = first year medical student; MS3 = third year medical student; R2 = second year internal medicine resident; F = experienced medical faculty.

For MS1’s, the number of coded sections focusing on *patient history* and *physical exam* were far less than those focusing on *tests*. For residents, *patient history* slightly outnumbered *tests*, and both were much greater than *physical exam*. For faculty, *patient history* elements far outnumbered both *physical exam* and *tests* (Figure 2). Coding to *cue generation* was greater for MS1s compared with PM, but coding to *experience* and *symptoms* was less. The ratio of *experience* to *cue generation* in MS1s was the lowest of all groups suggesting experience is being used to help formulate a diagnosis (data not shown).

Table 3 shows the results of answers to the trigger question “can you remember anyone with this disease” by learning level and memory type. MS1s relate far fewer episodic memories (instance scripts) compared with all other groups, including premedical students. Semantic memories increased with experience, particularly for the recall of prototypical examples.

**Table 3:** Classification of responses to the trigger question, “Can you remember anyone with this disease?”

Memory Episodes	PM	MS1	MS3	R2	F
**Episodic**	**20**	**4**	**20**	**21**	**18**
**Semantic**	**6**	**6**	**34**	**59**	**72**
Explanation	1	1	4	9	6
Prediction	0	0	5	10	3
Prototype	4	3	12	25	40
Qualifier	1	2	13	15	23

PM—premedical student; MS1—first year medical student; MS3—third year medical student; R2—second year internal medicine resident; F—faculty member.

On a scale from −3 (unpleasant) to +3 (pleasant), the emotional tone averaged 0.23 ± 0.26 (aroused) for PM, −0.07 ± 0.13 (tense) for MS1, 0.42 ± 0.31 (alert) for MS3, 0.51 ± 0.33 (aroused) for R2, and 0.78 ± 0.58 (excited) for F. MS1s were the only group that demonstrated an average negative affect. Interviews with MS1s suggested that this negative tone occurred because the volume of information to be learned was overwhelming, that retention was difficult, and that experience could not be trusted.

For instance:

**Example 6 **(MS1) “I’ll never be able to learn that much… but something that’s really helped, knowing people who have done it. I mean the fact that there are doctors, doctors exist, so it must be possible”.
**Example 7 (**MS1) “I may have seen cardiac enzymes but, you know, that kind of went in one ear and then came out on the exam”.
**Example 8** (MS1) “My exposure has been limited… although there is some, because… you know, I’ve-I’ve been told to listen for the crackling in the lungs… maybe it’s not crackling in the lungs, in which case it’s my [pause] misperception”.

Figure 2 above shows that, as experience grows, the importance of history for making a diagnosis increases.

### 3.2. Intermediates: Logistic Growth

**Figure 3:**
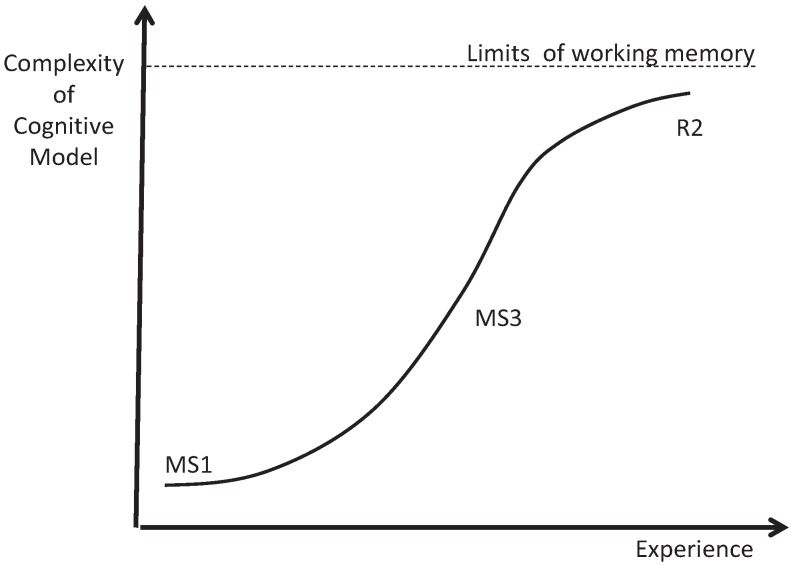
The logistic growth of diagnostic reasoning skills observed in intermediate learners.

**Figure A4:**
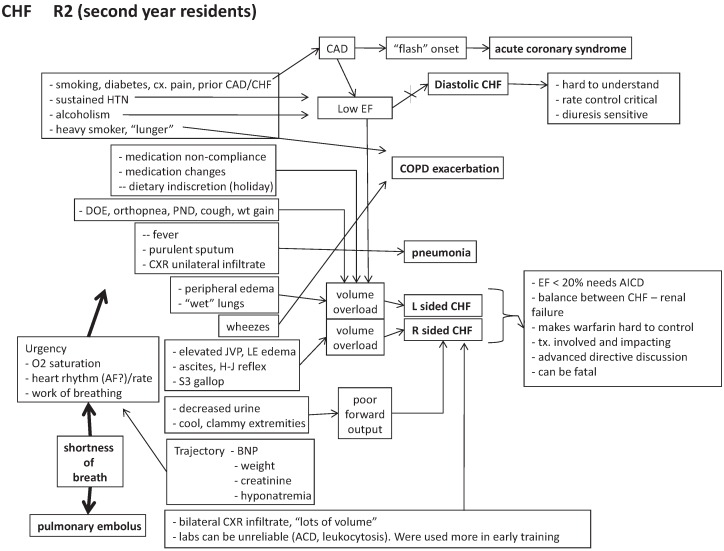
Second year medicine residents.

Our data show steep early growth in algorithmic diagnostic reasoning skills between the MS1 and MS3 years followed by slowing of growth, between the MS3 and R2 years (Figure 3). We observed this in the growth rate of concept connections (Table 1) and semantic memories (Table 3). It can also be seen as the steepest tangent on the propositional growth curve between the MS1 and MS3 years, with a slightly decreasing slop in all disease processes between the MS3 and R2 years in the proposition map (Figure 1). If, in fact, this growth is initially exponential it would be due to positive feedback. This positive feedback could be supplied by Hebb’s law [32], often stated as “neurons that fire together wire together”. The efficiency obtained from this reorganization would feed back on the handling of new and old information, and facilitate compilation into more efficient chunks. One piece of evidence from our study that suggests such conceptual chunking is the transition of “fluid buildup” (MS1/MS3—focused on concrete physical exam findings) to volume overload (R2/F—a more abstract concept that includes interactions between volume, vessel capacitance, and cardiac output) in the CHF concept maps. Logistic curves begin to flatten out as they reach a limit. We believe the limit in this case may be working memory, which can handle only roughly seven bits of information [33]. The cognitive maps of second year residents get too complex for the working memory to manage all the critical variables simultaneously (see Figure A4 in Appendix).

MS1 = First year medical students, MS3 = third year medical students, R2 = second year internal medicine residents.

### 3.3. Experts: Cognitive State Transition

**Figure A5:**
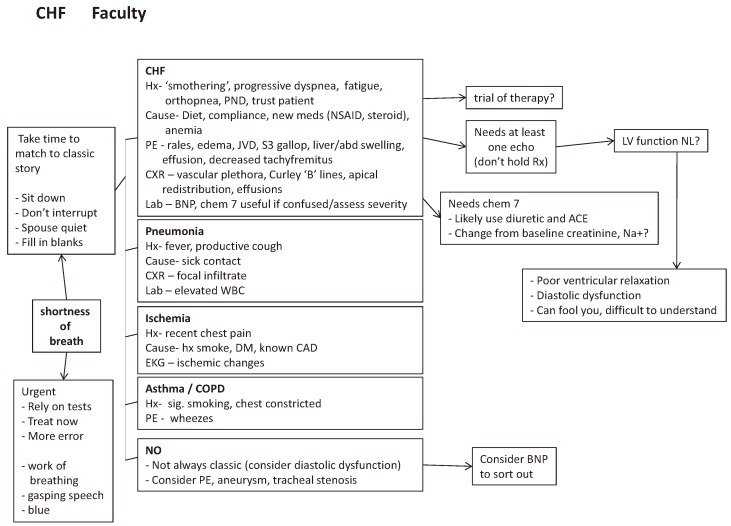
Experienced faculty.

In the propositional analysis, the average number of propositions per disease per training level peaked in the resident year. The word count per transcript steadily increased with experience level. Concept maps demonstrate a steady increase in complexity (as measured by concept connections) from first year students to residents, reflecting increasingly elaborated rule-based cognitive models (data not shown). The average number of propositions per disease group, percentage of total words that are propositions, and the number of concept map connections all show large drops between residents and faculty (Table 1 above). There is also a qualitative difference between residents and faculty in their concept maps (Appendix, Figure A4, residents and Figure A5, faculty). Residents have compiled their propositions into complex inferences, moving from signs and symptoms through pathophysiology to implications and plans (reminiscent of illness scripts [15]). Faculty concept maps are simpler and compiled around sorting into prototypical stories as the first step after assessing urgency.

**Figure 4:**
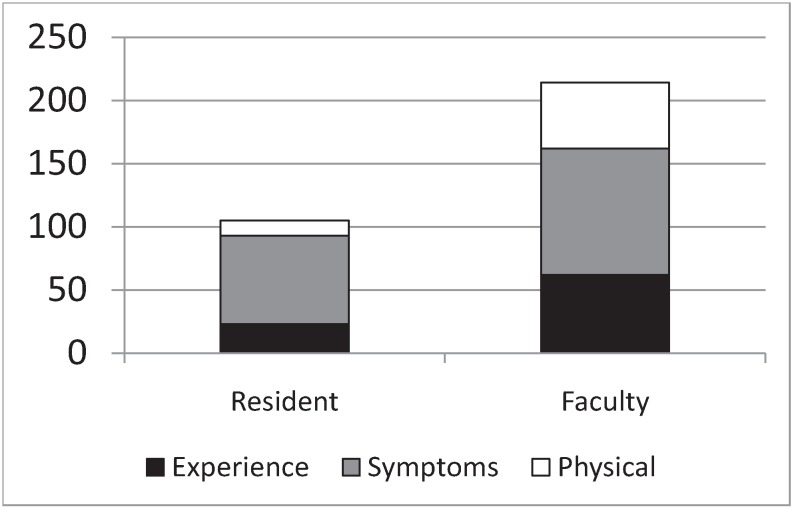
Average number of coded text units per transcript for residents and faculty members that were simultaneously coded as *cue generation* and *experience*, *symptoms*, or *physical exam.*

As seen previously in the grounded theory analysis (Table 2), there is a drop in the importance of tests and an increase in the use of disconfirming cues (“rule outs”) in the diagnostic process between residents and faculty. Figure 4 shows the average number of coding references per transcript for passages that were simultaneously coded as *cue generation* and three other salient categories; *experience*, *symptoms*, and *physical exam*.

The data in Figure 4 show that “cue generation” changes from largely symptom driven in residents to almost equally experience, symptom, and physical exam driven in faculty. Table 3 above shows the matrix of responses to the question “can you remember anyone with this disease”. The episodic (personal) memories of residents were generally the basis for the development of rules. Residents also had the highest rate of semantic (factual) memories scored as explanations and predictions. For instance, the following examples demonstrate the transition from episodes that imply rules to factual explanations that should be pursued.

**Example 9** (R2) “My last call, one patient had diastolic heart failure and had gained like 25 pounds, and they were not able to diurese him [as an outpatient]… The essential part here was to control his heart rate so the Lasix could start working.” (episodic memory supporting the rule *diastolic heart failure ➔ control heart rate*).
**Example 10** (R2) “I had a patient in clinic last week who presented with a weight gain of 25 pounds… (episodic memory) The classic is they have a trigger: Thanksgiving dinner, missing their medications, a small heart attack.” (semantic memory: explanations).

In contrast, the episodic memories of faculty members were often used to illustrate unfamiliar boundary cases and they often had specific semantic qualifiers representing ecological information (underlined). For instance:

**Example 11** (F) “My wife and I were having dinner at some friend’s house… She was a 26 year old woman who had twins about four weeks before and [we] were carrying on a conversation… something was bothering me… I looked over and realized she had Cheyne-Stokes breathing… Turns out she had post-partum congestive cardiomyopathy.” (episodic memory, unfamiliar cause of heart failure anchored to semantic qualifier for environmental information, Cheyne-Stokes breathing).
**Example 12** (F) “I was working in a small town, around 6000 [people], in the mountains. It was July, black fly season, and a woman came in with all the classic signs of pneumonia: cough, shortness of breath and fever and the classic physical findings, but it was the wrong season… I rechecked the chart and noticed she had Faget’s sign (pulse-temperature disparity)… she had Legionella.” (episodic memory, unfamiliar cause of pneumonia anchored to semantic qualifier for environmental information, Faget’s sign).

The purely semantic memories of faculty were often general prototypes that seemed preconscious (in that they had trouble coming up with the specific examples that supported these prototypes).

**Example 13** (F) “I haven’t had any really clear cut [cases of pneumonia] in the [last] two weeks. The ones that I admitted as a fellow are kinda blurring together into an archetype… you know, the person coming in without another explanatory cause who has fevers, respiratory symptoms, maybe some hypoxia, an infiltrate on their x-ray” (semantic memory: prototype).
**Example 14** (F) “Well, I see that in the clinic every week, I mean, it’s just numerous examples. I don’t remember a specific one… but they’re breathless by the time they get to your exam room, from the waiting room. I usually go out and get patients just ‘cause we have only one [exam] room and I typically have to walk slower and they’re pulling their oxygen bottle. Some of them kind of reek of cigarette smoke… I have a quite a few, [that are] pretty advanced, and typically they’re thin and, [the] spouse does most of the talking ‘cause the-patient gets breathless” (semantic memory: prototype).

Together, these data suggest that personal episodic memories for residents are the basis for potential rules. Over time, groups of rules are collated as prototypes and the individual case specifics are forgotten. However, for faculty the boundary cases that violate the prototype are again stored as episodic cases, which frequently include critical ecological information (important “rule ins” and “rule outs”).

Residents generate empirical cues primarily from the patient’s presenting symptoms (Figure 4). They are the most enthusiastic group regarding the value of tests (Figure 2). They describe the greatest number of propositonal rules when discussing diagnoses (Table 1), have the most robust concept maps (Appendix, Figure A4, residents), and have the richest most elaborated propositional structures (Table 1). Their episodic memories lead to working rules, and their semantic memories have the highest focus on explanations and predictions. Residents represent the pinnacle of a correspondence or representational model of truth [34]. Cognition is largely rule-based, analytical, and focused on management. Propositions correspond to real elements in the world. Categories are formal, with absolute boundaries and necessary and sufficient conditions. Memory is arranged hierarchically. Residents have technically detailed internal representations of disease, but these internal representations are very rigid. They have created highly elaborated conceptual systems, relying primarily on explicit memory, and use these for information processing to analytically prove a diagnosis. It is likely that the complexity of residents’ conceptual models begins to approach the limits of working memory. Working memory maintains representations of task-relevant information (perceptual data, thoughts, and memories) in order to regulate attention and support executive function (updating plans, task switching, and response selection). Working memory capacity is quite limited, handling only about seven “chunks” of information simultaneously [33], and it lasts seconds to minutes.

Faculty are qualitatively different. For entities commonly seen by faculty, they exhibit decreased complexity of their cognitive models and appear to rely more on intuition, agreeing with dual processing models [23,24]. They exhibit fewer propositions, lower complexity concept maps (see Appendix), and rely less on tests (Figure 2). Unlike residents, faculty members generate their diagnostic cues almost equally from experience, symptoms, and the physical examination (Figure 4). They have the highest ratio of semantic (factual) to episodic (personal specific) memories (Table 3) and their semantic memories focus on prototypes and qualifiers. Faculty members appear to rely on preconscious categorization, using fewer dimensions of information and processing it more crudely as all/none (examples 5 and 6 above). In areas that are less familiar, faculty seem to revert to the analytical approach and to store examples that are rich in relevant details as episodic memories (Examples 3 and 4 above).

This transition between resident and faculty appears to be a shift between two types of cognitive states; analytical and intuitive (Figure 4). Experienced faculty convert to a coherence model of truth in familiar areas [35], compiling their representations into a few core elements and quickly matching the patient’s presentation of illness to the best compiled story. Propositions focus on discriminating features that reliably separate diseases (such as disconfirming cues) and their concept maps contain default values for missing propositions. Prototypes are fuzzy, with a general central exemplar and a few specific high-detail cases as you get near a boundary. Memory is arranged relationally, in groups based on similarity.

**Figure 5:**
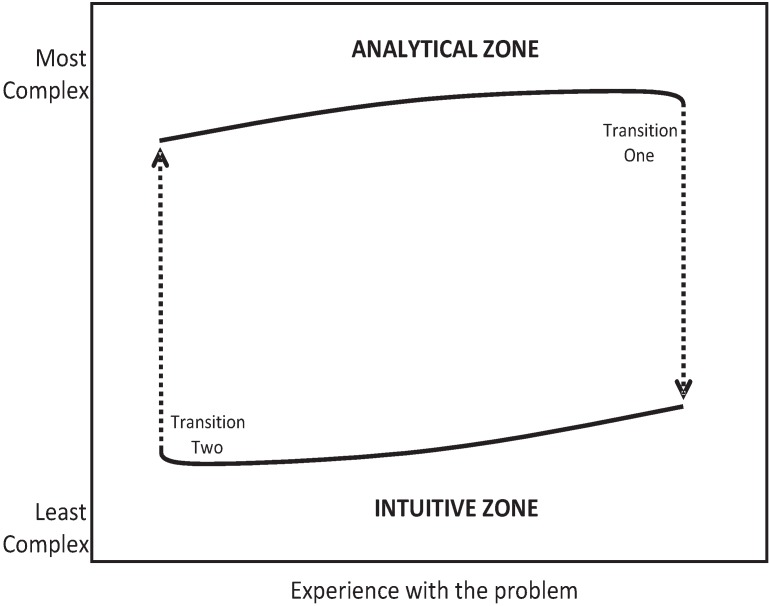
Zone map comparing complexity of the conceptual model *versus* experience.

**Figure 6:**
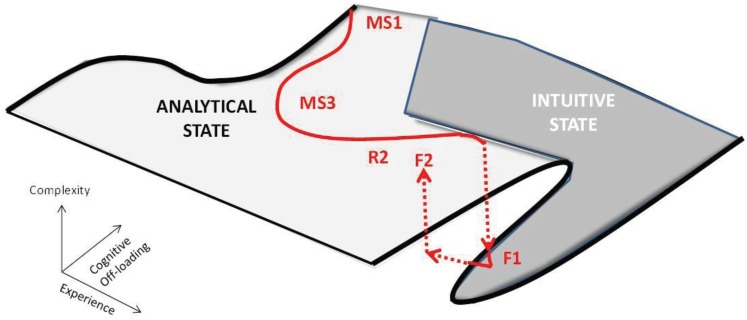
The cognitive landscape showing a critical fold, typical learner trajectory (in red) and a critical state change that occurs with combinations of experience and cognitive off-loading.

This change from resident to experienced faculty may represent a critical state transition between alternative stable states (Figure 5) [36], akin to H_2_O going from a solid to a liquid as the temperature rises. The new substance is from the same basic material but is qualitatively very different. This would agree with dual processing theories of clinical reasoning [23,24]. Complexity science posits the possibility of alternative stable states in dynamical systems. Analytical and intuitive approaches may represent just such a state transition. Signs include: (1) sharp transitions; (2) a decreasing rate of change as you approach transitions; and (3) transitions in opposite directions occurring at different levels of experience (hysteresis) [36]. Alternative stable states typically require positive feedback, like a pencil balancing on its point, where any perturbation is amplified [36]. In the case of cognitive models, the positive feedback as stated previously could be supplied by Hebb’s law [32], often stated as “neurons that fire together wire together”. Recurrent experience with common problems could lead to more efficient, self-reinforcing cognitive models. This could happen in two ways. First, common scenarios could develop into preconscious scripts, schemas and other narrative structures that require less working memory than analytical versions. Second, ecological cognition could replace information in cognitive models with information expected in the patient and the environment. Experienced faculty use both in order to off-load working memory. Faculty have the highest semantic to episodic memory ratio and tend to remember common problems as general prototypes (examples 13 and 14). Unlike all other groups, faculty concept maps begin with sorting the presentation into the best matched story. Also, faculty tacitly scan the environment for relevant information. While they probably do this frequently, it was identified in our data when they had reverted to an analytical approach in unfamiliar areas (examples 11 and 12). Both of these methods of cognitive off-loading could result in the decreased complexity we observed and would agree with extended, ecological cognition. Figure 6 shows a cognitive landscape in three dimensions (experience, complexity, and cognitive off-loading) with the typical learner trajectory. Note that between the resident and faculty (F1) levels there is a critical state transition (from analytical to intuitive). If faculty are faced with problems that are unfamiliar enough, they revert back to an analytical state (F2). This model, which represents initial analytical processing followed by cognitive off-loading using dual processing and ecological cognition, fits our data well.

MS1 = first year medical student; MS3 = third year medical student; R2 = second year internal medicine resident; F = experienced faculty.

The state transition theory would explain several of the anomalies mentioned earlier. Residents would be expected to have the richest propositional structure just before state transition (the intermediate effect). Experts would start with better hypotheses based on promising-primed ecological intuitions. Because this process is preconscious, experts may have trouble describing in propositional structure how diagnoses were made. Like the fire chief mentioned earlier, they may require extended guided reflection to discover their tacit propositional rules. Experts would jump up from the lower curve and back into an analytic propositional state (Figure 5) in areas where their experience was low.

There are some limitations of our study. It was largely conducted in a single academic training system. For some participants, especially residents and faculty, some of the researchers were known, while for other participants the protocol may have seemed more like a test administered by strangers. This might result in a differential motivation to participate. Our methods largely reflect an academic format—questions about how to approach a diagnosis—and this may accentuate the differences we found. For some participants (primarily residents, due to outside rotations), data collection spanned a few months and this may have contributed to skill heterogeneity.

## 4. Conclusions

In this study we confirm the importance of patient history for diagnosis and have supported many elements of the encapsulation theory. We also identified an apparent discontinuity for MS1s (novices) in the expected development of diagnostic expertise, which we call the “apprentice effect”. This appears to be due to barriers to accessing knowledge due to anxiety and embarrassment. This is manifested by emotional stress, cognitive overload, mistrust of experience, narrative sparseness (a drop in the number of propositional assertions and concepts), and misplaced concreteness compared with premeds, MS3s, or residents.

Our findings suggest that a curriculum with decreased memorization, safe exploration of speculative ideas based on experience (whether right or wrong), and early exposure to prototypical clinical cases might better facilitate the learning process and development of diagnostic expertise in early medical students. This study was carried out in an institution with a traditional curriculum of two years of basic sciences followed by two years of clinical training. This type of curriculum may accentuate the apprentice effect in the first year by dissociating the learning of facts during didactics from their clinical applicability. It would be important to see if the apprentice effect is ameliorated in schools using an early problem-based or team-based learning approach.

Intermediate learners appear to gain algorithmic diagnostic reasoning skills following a logistic pattern. The positive feedback loop required for this behavior may be due to Hebb’s law (neurons that fire together wire together), suggesting again that sufficient exposure to the normal variance of prototypical cases is important. The limit that is reached with this mode of reasoning may be working memory. Foreshadowing this phenomenon may be important for intermediate learners.

Experts demonstrate a potential state transition in diagnostic reasoning from rule-based correspondence, classical categories, and hierarchical memory structures (residents) to story-matching coherence, fuzzy categories, relational memory structures, using promising-primed ecological cognition (faculty). Further research to confirm the tacit narrative structures in intuition and subconscious perceptual scanning associated with ecological cognition in experts would help to elucidate elements that may have rich educational and error reduction implications.

This study supports the encapsulation theory of learning for novices through intermediate level learners. During these periods, learners should be exposed to integrated didactic and clinical curricula for maximal benefit. The theory of recognition-primed decision making best explains the diagnostic reasoning of experts in their domain of expertise. Experts revert to analytical reasoning when diseases are less familiar. Advanced trainees would benefit from attention to intuitions, with feedback about their intuition’s accuracy. Although there are hints that the theory of ecological psychology may be important for understanding tacit elements of expertise, our study methods could not confirm this. Comparing residents and faculty in real time natural observations of diagnosis, with attention to elements such as sympathetic tone, direction of gaze, and saccadic eye movements, may be important for understanding the process of diagnosis (and making diagnosis error) and should be further studied.

## Figures and Tables

**Figure A2:**
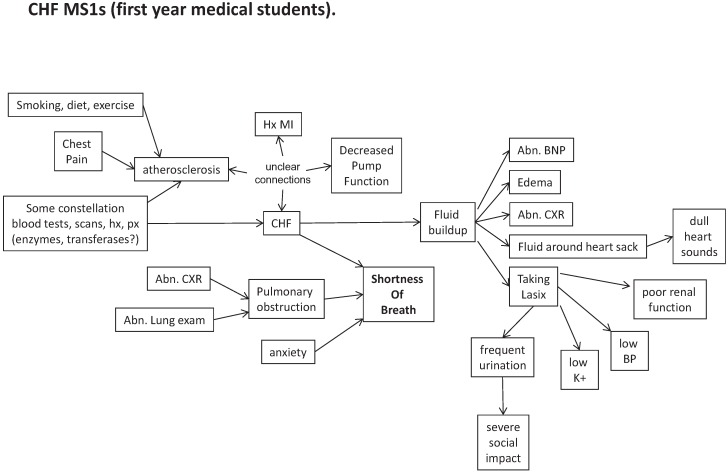
First year medical students.

**Figure A3:**
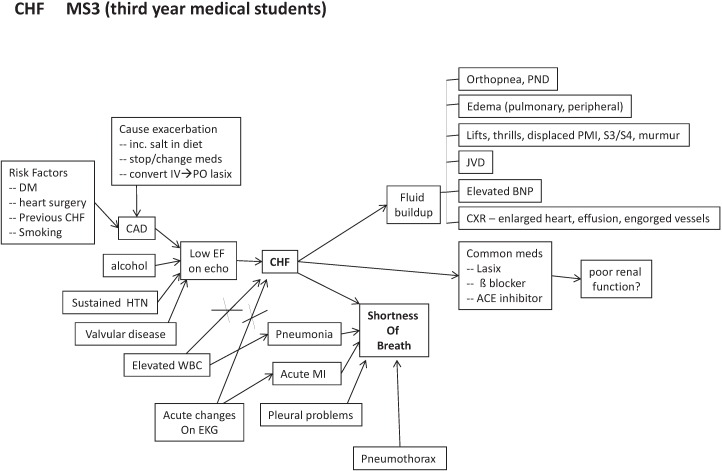
Third year medical students.

## Appendix

Concept maps of “CHF” for each training level.
